# Inherited IL-18BP deficiency in two Egyptian siblings with fulminant viral hepatitis

**DOI:** 10.70962/jhi.20250135

**Published:** 2025-09-26

**Authors:** Dalia Abd Elaziz, Alperen Baran, Aysima Atilgan Lulecioglu, Adrian Gervais, Chenglin Zhang, Vivien Béziat, Xiao-Fei Kong, Hanaa El-Karaksy, Jean-Laurent Casanova, Serkan Belkaya, Emmanuelle Jouanguy

**Affiliations:** 1 Pediatrics Department, Faculty of Medicine, Cairo University, Cairo, Egypt; 2 Alder Hey Children’s NHS Foundation Trust, Liverpool, UK; 3 Department of Molecular Biology and Genetics, Faculty of Science, İhsan Doğramacı Bilkent University, Ankara, Turkey; 4 Laboratory of Human Genetics of Infectious Diseases, Necker Branch, INSERM U1163, Paris, France; 5 Paris Cité University, Imagine Institute, Paris, France; 6 Laboratory of Human Genetics of Infectious Diseases, Rockefeller Branch, Rockefeller University, New York, NY, USA; 7 Department of Internal Medicine, UT Southwestern Medical Center, Dallas, TX, USA; 8 Pediatric Hepatology Unit, Faculty of Medicine, Cairo University, Cairo, Egypt; 9 Department of Pediatrics, Necker Hospital for Sick Children, Paris, France; 10 Howard Hughes Medical Institute, New York, NY, USA

## Abstract

A previous report described an inherited deficiency of IL-18BP, a soluble antagonist of IL-18, in an Algerian patient who died of fulminant viral hepatitis (FVH) A. We report here an Egyptian family with two siblings who died from FVH following infection with hepatitis A virus.

## Introduction

Fulminant viral hepatitis (FVH) is a rare, mostly sporadic, life-threatening disease characterized by massive liver destruction, typically following infection with hepatitis A or B virus (HAV, HBV). In rare cases, it can be triggered by herpes simplex viruses. FVH usually affects otherwise healthy individuals and occurs at an estimated annual incidence of one to five per million infected individuals. FVH outcome remains poor, regardless of the trigger virus, with survival rate below 50% without liver transplantation. FVH is not epidemic or linked to specific viral genotypes (1), making it unlikely to be triggered by more virulent viral strains. While typically sporadic, three familial clusters of FVH have been reported, with intervals of years between successive cases, suggesting that FVH may result from monogenic inborn errors of liver immunity to viruses, with incomplete penetrance, at least in some patients. Supporting this hypothesis, we recently reported autosomal recessive (AR) IL-18BP and IL-10RB deficiencies as the first genetic etiologies of FVH following HAV infection (2, 3). IL-10, IL-10RA, and IL-10RB deficiencies are known genetic causes of early-onset inflammatory bowel disease (EOIBD). Two Saudi siblings homozygous for deleterious *IL10RB* variants with EOIBD died of HAV-induced FVH at 6 and 1 years of age. Patients with complete IL-10RB deficiency display impaired cellular responses to IL-10, IL-22, IL-26, and IL-28/29 (also known as type III IFNs). By contrast, these two siblings had a partial form of IL-10RB deficiency, restricted to the abolition of cellular responses to IL-10 (3). Impaired IL-10 immunity therefore underlies not only EOIBD, as deduced from the EOIBD phenotype of IL-10– and IL-10RA–deficient patients, but also FVH, as inferred from these two IL-10RB–deficient siblings. Since IL-10 is a major anti-inflammatory cytokine and antagonist of the macrophage-activating factor IFN-γ, uncontrolled inflammation might be involved in FVH pathogenesis. IL-18BP deficiency was first reported in a previously healthy Algerian patient who died of HAV-FVH at age 11 (2). IL-18BP, mainly secreted by macrophages, antagonizes IL-18, a pleiotropic cytokine mostly produced by hematopoietic cells, such as monocytes, macrophages, and dendritic cells, but also by endothelial and epithelial cells, including hepatocytes. IFN-γ upregulates IL-18BP and downregulates IL-18, these two effects converging to reduce inflammation. In the patient with IL-18BP deficiency, the lack of IL-18BP unleashed IL-18. The potential consequences of excessive IL-18 activity include increases in blood IFN-γ levels. It has been suggested that enhanced IFN-γ production drives FVH, as this mechanism could account for the uncontrolled macrophage activation in the HAV-infected liver (2). Moreover, it is tempting to speculate that excessive IFN-γ activity—whether due to a deficiency of the IFN-γ-antagonist IL-10 or an excess of the IFN-γ–inducing IL-18—is a potentially pathogenic feature common to both IL-10RB and IL-18BP deficiencies. In this context, we studied two Egyptian siblings with FVH following HAV infection.

## Case report

### Homozygosity for a private frameshift mutation in IL18BP

**Figure 1.**
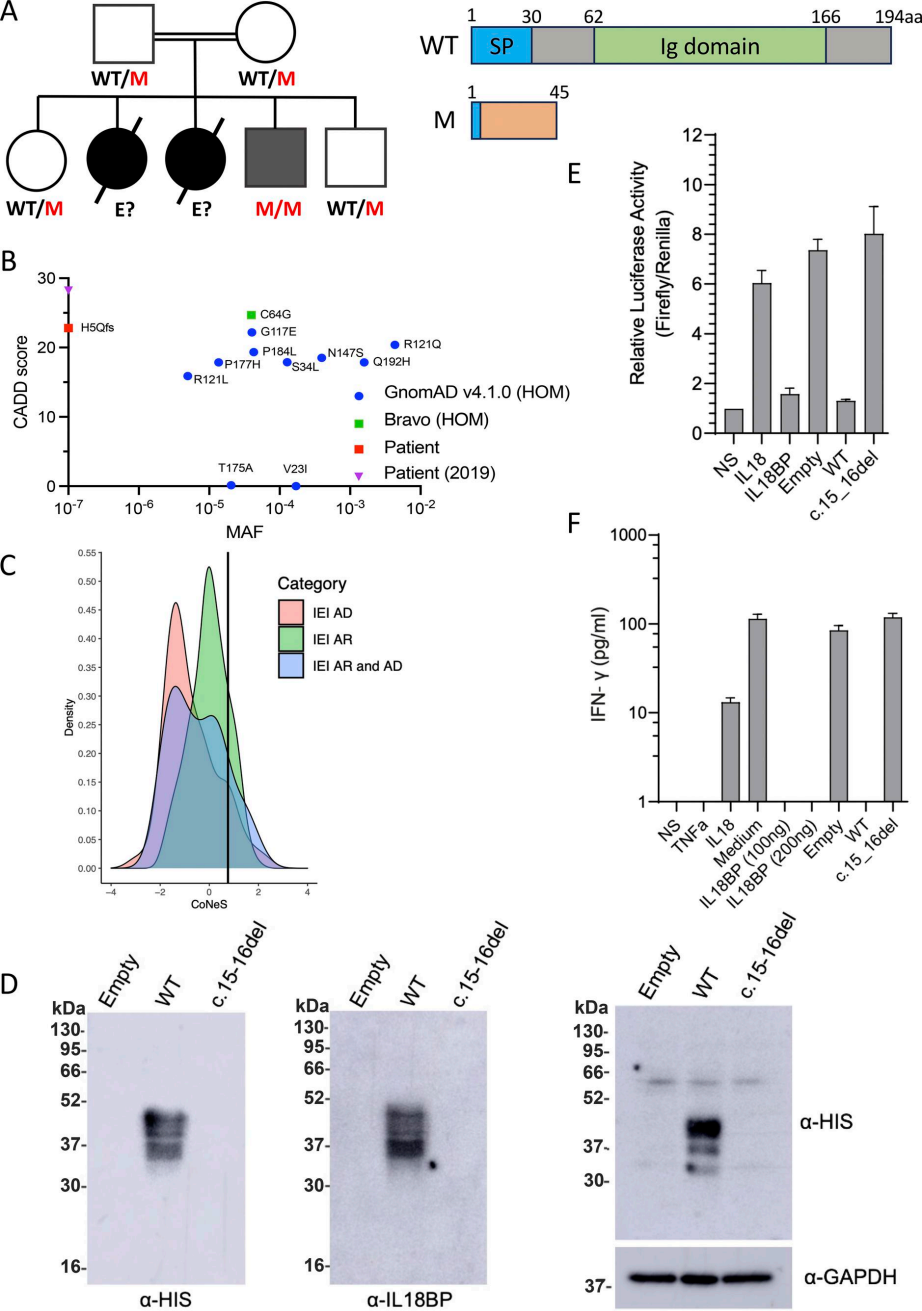
**IL18BP c.15-16del is loss-of-function.** **(A)** Pedigree and familial segregation of the mutations and schematic representation of IL-18BP. **(B)** CADD vs. MAF graph for homozygous *IL18BP* variants from gnomAD and Bravo and the patients’ allele. **(C)** CoNeS score of IL-18BP. **(D)** Representative western blot for secreted IL-18BP (His-tag) in non-concentrated supernatant (10 μg protein/lane) (left) and for intracellular IL-18BP (His-tag) in lysates (20 μg protein/lane) (right) from transiently transfected COS7 cells. Data are representative of five independent experiments. **(E)** Relative luciferase activity (Firefly to *Renilla* signal ratio) in HEK293 cells transiently transfected with NF-κB–Firefly luciferase + IL-18RAP + *Renilla* luciferase plasmids upon 12-h stimulation with recombinant IL-18 (5 ng/ml) and/or recombinant WT IL-18BP-His (100 ng/ml) or concentrated supernatant (100 μg/ml of total protein) from COS7 cells transiently transfected with empty, WT, or mutant IL-18BP–tagged constructs. Relative luciferase activity was normalized against non-stimulated (NS) cells, for which the value was set to 1. **(F)** IFN-γ production detected by ELISA on the supernatant of KG-1 cells stimulated for 24 h with recombinant TNF (20 ng/ml), recombinant IL-18 (20 ng/ml), and/or recombinant WT IL-18BP-His (100–200 ng/ml) or concentrated supernatant (175 μg/ml of total protein) from COS7 cells transiently transfected with empty, WT, or mutant IL-18BP–tagged constructs. **(E and F)** Data are presented as the mean ± SEM of five independent experiments. All IL-18BP constructs and supernatants were generated as previously described (2). WT, wild-type; M, mutated; E?, unknown genotype; SP, Signal peptide; P, patient; MAF, minor allele frequency; HOM, homozygous; IEI, Inborn errors of Immunity; AD, autosomal dominant. Source data are available for this figure: SourceData F1.

Two children born to Egyptian first-cousin parents developed acute liver failure. The index case (P1) died at 2 years of age on the first day of hospitalization in 2016. The second child (P2) died of acute liver failure at 7 mo of age after 7 days of hospitalization in 2017. Both tested positive for anti-HAV IgM. A third child (P3) developed self-healing hepatitis at 22 mo of age in 2022, with high levels of transaminases but no jaundice or encephalopathy. He tested negative for anti-HAV IgM but positive for CMV IgM and EBV-VCA IgM. P3 was not hospitalized and recovered without antiviral therapy. He was subsequently vaccinated against HAV after the genetic diagnosis and against HBV as a part of compulsory vaccination schedule in Egypt with three doses starting at birth (0, 1, and 6 mo of age) and has remained healthy until his most recent follow-up visit at the age of 5 years in 2025. Whole-exome sequencing on P3 detected homozygosity for a frameshift (c.15_16del) mutation in *IL18BP* leading to a premature stop codon (p.His5Glnfs*40). This variant was not reported in any public databases (1000 Genomes, single nucleotide polymorphism database []dbSNP, gnomADv4.1, or Bravo) or our in-house database of >25,000 exomes. The familial segregation of the mutant *IL18BP* allele was consistent with an AR mode of inheritance with complete penetrance, as both parents and two healthy siblings were heterozygous for the variant (Fig. 1 A). The two parents and the two healthy siblings had been vaccinated against HAV, and the two siblings against HBV. No genetic testing could be performed for P1 and P2, due to a lack of material. In silico predictions for the *IL18BP* variant suggested that it would be highly deleterious, with a combined annotation-dependent depletion (CADD) score of 22.8, above the mutation significance cutoff of 12.2 for *IL18BP* (Fig. 1 B). *IL18BP* has a gene damage index of 4.003 and a consensus negative selection (CoNeS) score of 0.773, consistent with an AR trait (Fig. 1 C). Only 11 variants of this gene have been reported in the homozygous state in gnomADv4.1 and Bravo, and all these variants are missense, with an allele frequency in the general population between 1.5 × 10^−5^ and 4.3 × 10^−3^. Collectively, these findings suggest that homozygosity for the probably deleterious private allele c.15_16del of *IL18BP* may have contributed to CMV/EBV hepatitis in P3 and probably to HAV-FVH in his two siblings.

### The patients’ IL18BP allele is loss-of-function (LOF)

We generated His-tagged cDNA constructs for expression of the IL-18BP frameshift variant (IL-18BPfs) as described (2), to assess its impact on protein production in vitro. In a transient overexpression system in COS-7 cells, no IL-18BPfs protein was detected in either the supernatant and cell lysate, contrasting with the findings for IL-18BP-WT (Fig. 1 D). We then investigated the functional impact of the variant with a luciferase assay in the HEK293T cell line in vitro. HEK293 cells transiently transfected with NF-κB–Firefly luciferase, *Renilla* luciferase, and a vector expressing IL-18RAP were stimulated with IL-18 either alone or in combination with the supernatants of COS7 cells transiently transfected with empty vector, IL-18BP-WT, or IL-18BPfs. Levels of IL-18–induced NF-κB activity were comparable between the supernatants of cells transfected with empty vector or IL-18BPfs and similar to those obtained with IL-18 alone (Fig. 1 E). We also measured IFN-γ production by KG-1 macrophages following TNF/IL-18 costimulation, with or without supernatants of COS7 cells transfected with empty vector, WT, or IL-18BPfs constructs. No IL-18 inhibition was observed with the mutant IL-18BP relative to the WT, as previously documented with the pathogenic c.508-19_528del LOF allele found in the homozygous state in an Algerian patient with FVH (Fig. 1 F) (2). The c.15_16del allele results in a complete absence of IL-18BP and a lack of IL-18–neutralizing activity. These findings suggest that P3 has AR IL-18BP deficiency, which was also probably the cause of HAV-FVH in P1 and P2.

## Discussion

We report a second family with FVH triggered by HAV in two children and self-healing hepatitis triggered by CMV/EBV in a third child from the same family, due to preexisting, inherited complete IL-18BP deficiency. This observation confirms that AR IL-18BP deficiency is a genetic etiology of HAV-FVH in otherwise healthy children normally resistant to other microbes. The same phenotype was seen in children from two families from different countries (Algeria and Egypt) homozygous for different LOF variants (c.508-19_528del and c.15_16del) (2). None of the 11 variants found in the homozygous state in public databases are predicted LOF, implying that AR IL-18BP deficiency is exceedingly rare. AR IL-18BP should be considered in other patients with FVH. Interestingly, while the Algerian and two Egyptian siblings died of HAV FVH, the third Egyptian child recovered from hepatitis triggered by CMV and EBV, implying that IL-18BP is more important for the control of HAV than for the control of herpes viruses. IL-18BP may therefore be considered essential and nonredundant for the control of hepatic inflammation during infection of the liver by HAV. It is otherwise remarkably redundant for defenses against common microbes. Indeed, despite their young age, none of the three Egyptian siblings developed any other clinical phenotype after infection with other common viruses or other microbes, as previously reported for the Algerian child (2). In conclusion, inherited IL-18BP deficiency is a genetic etiology of HAV-FVH in otherwise healthy children. Measuring IL-18BP levels in clinical practice would improve diagnosis and guide treatment, since recombinant human IL-18BP is already available and used in other various inflammation conditions (4, 5).

## Supplementary Material

SourceData F1 is the source file for Fig. 1.

## References

[1] Jouanguy, E. 2020. Human genetic basis of fulminant viral hepatitis. Hum. Genet. 139:877–884. 10.1007/s00439-020-02166-y 32285199 PMC7153696

[2] Belkaya, S., E. Michailidis, C.B. Korol, M. Kabbani, A. Cobat, P. Bastard, Y.S. Lee, N. Hernandez, S. Drutman, Y.P. de Jong, . 2019. Inherited IL-18BP deficiency in human fulminant viral hepatitis. J. Exp. Med. 216:1777–1790. 10.1084/jem.20190669 31213488 PMC6683989

[3] Korol, C.B., S. Belkaya, F. Alsohime, L. Lorenzo, S. Boisson-Dupuis, J. Brancale, A.L. Neehus, S. Vilarinho, A. Zobaida, R. Halwani, . 2023. Fulminant viral hepatitis in two siblings with inherited IL-10RB deficiency. J. Clin. Immunol. 43:406–420. 10.1007/s10875-022-01376-5 36308662 PMC9892130

[4] Geerlinks, A.V., A.M. Dvorak, and XIAP Deficiency Treatment Consortium. 2022. A case of XIAP deficiency successfully Managed with Tadekinig Alfa (rhIL-18BP). J. Clin. Immunol. 42:901–903. 10.1007/s10875-022-01236-2 35304666

[5] Canna, S.W., C. Girard, L. Malle, A. de Jesus, N. Romberg, J. Kelsen, L.F. Surrey, P. Russo, A. Sleight, E. Schiffrin, . 2017. Life-threatening NLRC4-associated hyperinflammation successfully treated with IL-18 inhibition. J. Allergy Clin. Immunol. 139:1698–1701. 10.1016/j.jaci.2016.10.022 27876626 PMC5846100

