# Supplementary material for: Inherited IL-18BP deficiency in two Egyptian siblings with fulminant viral hepatitis
Source: J Hum Immun. 2025 Sep 26;1(4):e20250135. doi: 10.70962/jhi.20250135 (PMC12668466; doi:10.70962/jhi.20250135)
Supplement: SourceData F1 — is the source file for Fig. 1. [file jhi_20250135_sourcedataf1.pdf]

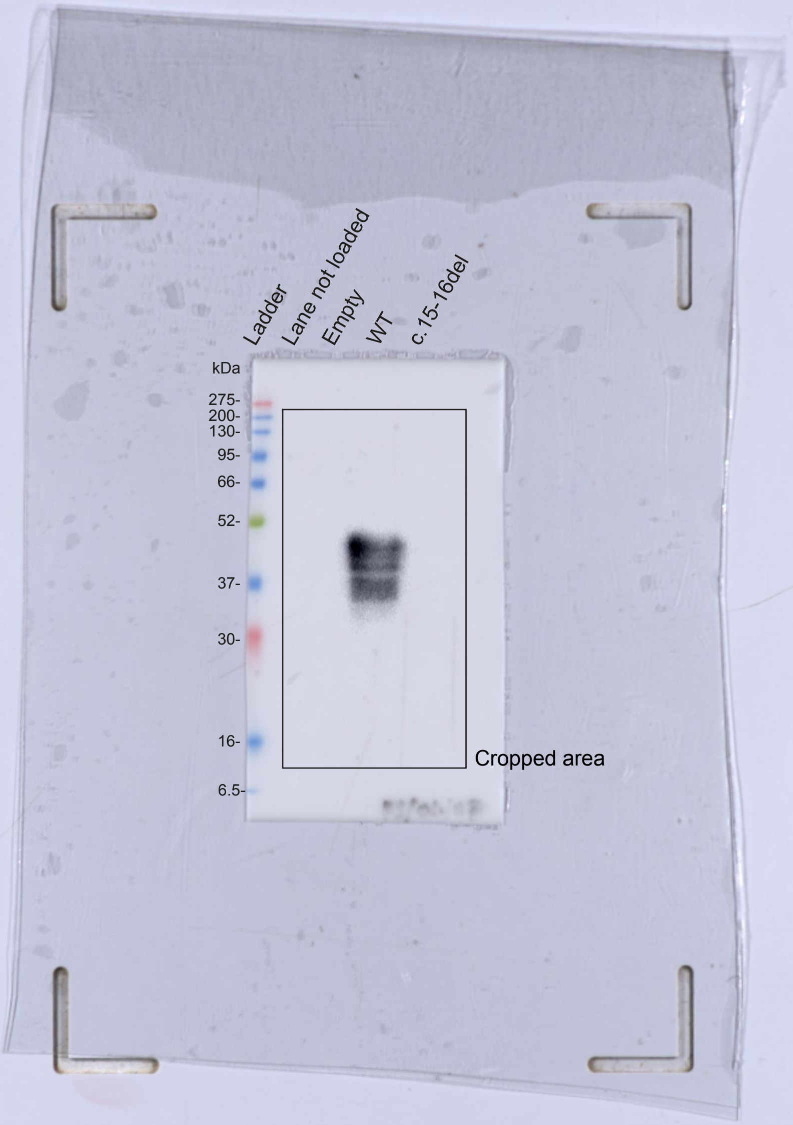
Ladder
Lane not loaded
Empty
WT
c.15-16del
kDa
275-
200-
130-
95-
66-
52-
37-
30-
16-
6.5-
Cropped area

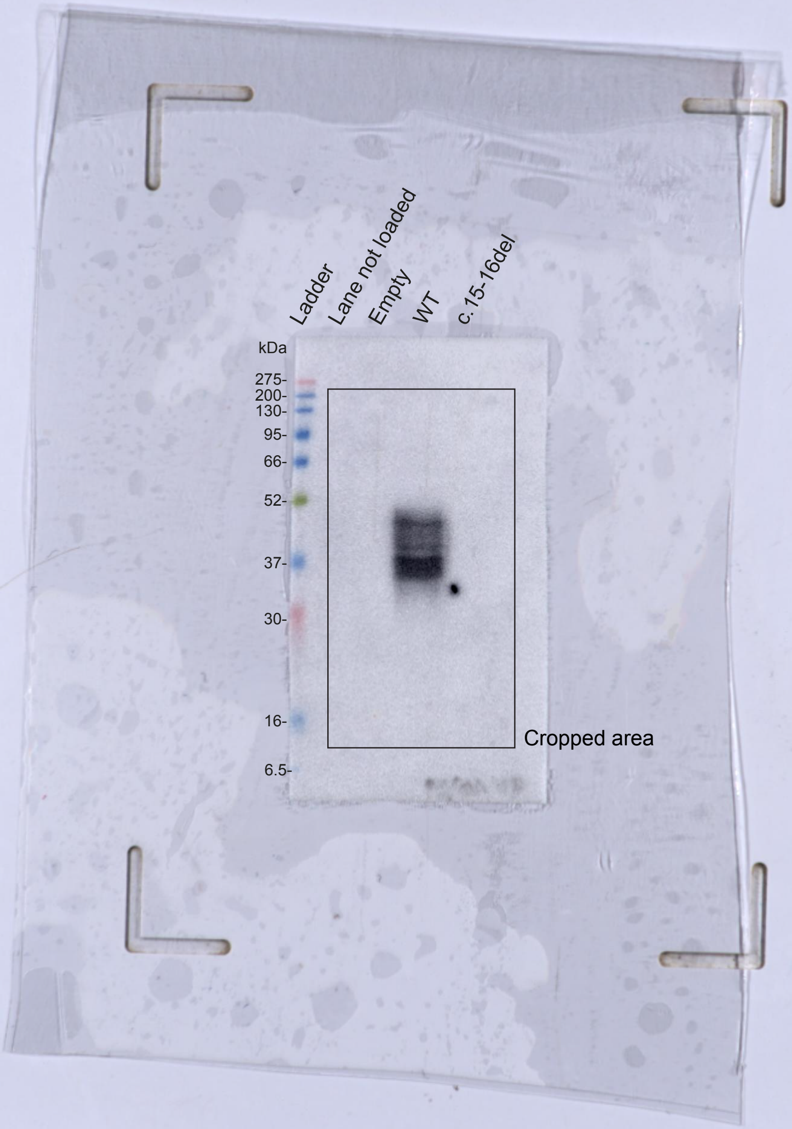
Ladder
Lane not loaded
Empty
WT
c.15-16del
kDa
275-
200-
130-
95-
66-
52-
37-
30-
16-
6.5-
Cropped area

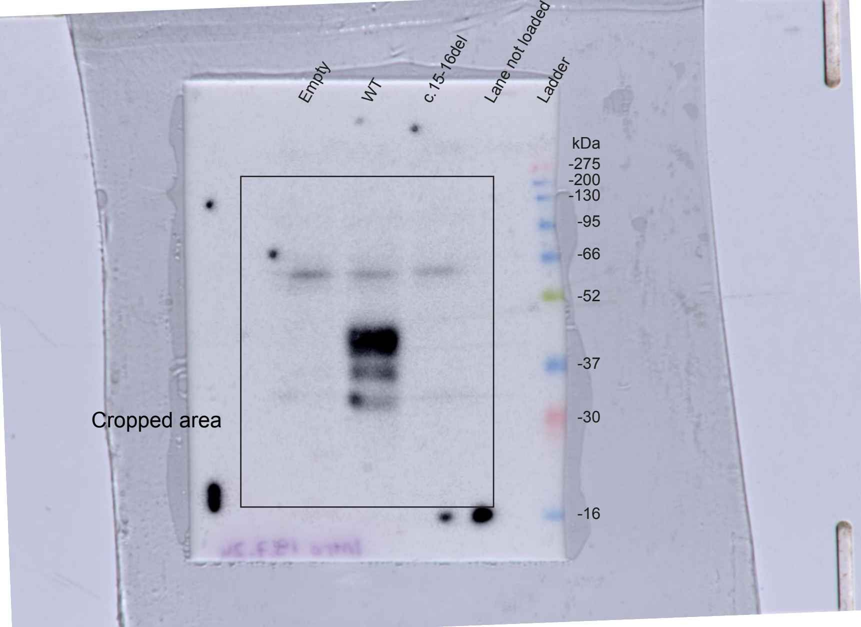

Empty
WT
c.15-16del
Lane not loaded
Ladder
kDa
-275
-200
-130
-95
-66
-52
-37
-30
-16
Cropped area

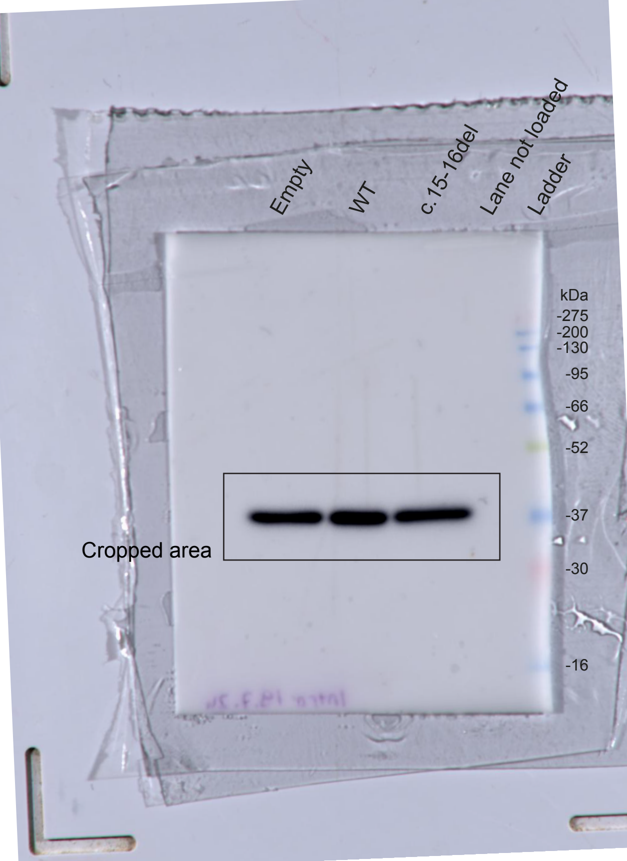
Empty
WT
c.15-16del
Lane not loaded
Ladder
kDa
-275
-200
-130
-95
-66
-52
-37
-30
-16
Cropped area
